# Factors Affecting Body Image Distortion in Adolescents

**DOI:** 10.3390/children9121944

**Published:** 2022-12-11

**Authors:** Eun-Ha Jung, Mi-Kyoung Jun

**Affiliations:** 1 Department of Dental Hygiene, College of Medical Convergence, Catholic Kwandong University, Gangneung 25601, Republic of Korea; 2 Department of Dental Hygiene, Dongnam Health University, Suwon 16328, Republic of Korea

**Keywords:** adolescent, body image distortion, body mass index, health behavior

## Abstract

Body image distortion is an important problem in physical and psychological health in adolescents. This study examined the factors affecting body image distortion in Korean adolescents. A Rao–Scott χ^2^ test and a complex samples logistic regression was conducted using the statistics from the 17th (2021) Korea Youth Risk Behavior Web-based Survey. The study sample included 41,124 middle and high school students. There was a difference in the presence or absence of body image distortion according to the subject’s gender, school grade, school achievement, and economic status (*p* < 0.001). Approximately 49.5% of subjects with body image distortion had tried to lose weight, but there were few cases where they attempted to lose weight through physical activities, such as moderate physical activity (22.8%), vigorous physical activity (23.3%), and muscle physical activity (23.9%) (*p* < 0.001). The group with body image distortion was 1.77 times more likely to sit for more than four hours a day on average (*p* < 0.001). To prevent various factors influencing the distortion of the adolescent’s body image, the development of a systematic intervention program for forming no distortion of adolescents’ body image is required.

## 1. Introduction

Body image is the subjective view individuals have of their own body, regardless of how their body actually looks. Body image is a complex construct comprising thoughts, feelings, evaluations, and behaviors related to one’s body, and it keeps changing throughout life [1,2]. Body image is formed mainly in adolescence. During adolescence, there is a tendency to recognize one’s body type subjectively, and a body image is produced through social comparison, which evaluates the appearance of oneself while comparing it with others [3,4]. In particular, as ‘lookism’ expands, the number of adolescents who perceive their body image negatively is increasing because of the evaluation of their appearance by others, such as parents or friends, and exposure to excessively skinny and unrealistic body images depicted in various media [3,5].

Body dissatisfaction refers to the difference between one’s perceived body and the ideal body. Body image distortion is the difference between one’s perceived and actual body image. The incidence rate of body image distortion in adolescents is high. According to previous studies, 51.8% of adolescents have body image distortion, so it is necessary to pay more attention to adolescents with body image distortion [6]. In adolescents, negative body image is associated with mental health, such as stress, anxiety, depression, and eating disorders. In addition, a more negative body image can result in unhealthier eating behaviors, such as an increase in the number of meals, overeating, or restrictive eating [7]. In addition, body image distortion affects health behavior practices, such as physical activity participation [8]. Therefore, in the case of body image distortion, it is necessary to understand the occurrence of body image distortion and form a healthy body image in adolescence because it can negatively affect the whole body and mental health of adolescents.

Therefore, this study was intended to identify factors that can be associated with body image distortion.

## 2. Materials and Methods

### 2.1. Study Design

This cross-sectional study is a secondary analysis of descriptive research using the 17th Korea Youth Risk Behavior Web-based Survey (KYRBS, 2021). The data were analyzed to find the factors associated with body image distortion. The STROBE guideline was used to evaluate the quality of reporting for cross-sectional studies.

### 2.2. Participants

The Adolescent Health Behavior Online Survey has been conducted annually by the Korea Disease Control and Prevention Agency (KCDC). It is an anonymous, self-reported online survey targeting middle school and high school students. As of April 2021, students enrolled in middle and high schools nationwide were the population, and data collection was carried out through the stages of population stratification, sample distribution, and sampling. In the population stratification stage, 39 regional groups and school levels (middle school, general high school, and specialized high school) were used as stratification variables. As a result, the population was divided into 117 layers. The number of sample schools was then distributed by applying the proportional distribution method. According to the stratified cluster sampling method, the primary sampling unit was the school, and the secondary sampling unit was the class. Finally, a total of 54,848 adolescents (92.9%) participated in this survey (399 middle schools and 397 high schools).

To check the distortion of the body image according to the index definition of the 2017 Adolescent Health Behavior Online Survey Raw Data Usage Guidelines [9], the 85th percentile of normal weight or underweight, referred to as the body mass index (BMI) by age [10] of the 2007 growth chart for children and adolescents, 41,124 adolescents with fewer than the number were finally analyzed. This study was approved for exemption from deliberation by the Bioethics Committee of a public institution (NO: P01-202208-01-027, date of approval 17 August 2022). The KYRBW was downloaded from the website of the KCDC after approval for the use of raw data.

### 2.3. Instruments

The 17th KYRBWS consisted of an anonymous self-report questionnaire with 16 areas and 109 questions. In this study, the related questions were extracted and reclassified according to the study purpose by extracting the subjects’ socio-demographic characteristics (five items), BMI and weight control (three items), physical activity (three items), sitting down behavior and smartphone use (two items).

#### 2.3.1. General Characteristics

Gender, grade, school achievement, economic status, and residential type were included as general characteristics. The grades were divided into middle and high school. The school achievement was classified into ‘high, middle high, middle, middle low, low’ as the answers to the questions about academic performance in the last year. The economic status was classified into ‘high, middle high, middle, middle low, low’ according to the responses to the questions asking about the economic status of the household as perceived by the person. The type of residence was divided into ‘family, relative’s house, lodging, dormitory, and childcare facility’.

#### 2.3.2. Body Image Distortion

In this study, body image distortion was judged using the BMI and responses to one’s subjective body image recognition question (‘what do you think of your body shape?’). Before analysis, the BMI was calculated as the weight (kg)/height (m^2^) using the data collected from adolescents. The calculated BMI was divided into two levels (low weight and normal). When the actual weight of the study subjects was underweight (under the 5th percentile of the BMI), and their body type responded with ‘normal’, slightly oversized’, and ‘very oversized’ and the actual weight of those who responded that their body type was ‘little oversized’ or ‘very oversized’, they were classified as ‘distorted body image’ despite this normal weight (a body mass index in the 5th–84th percentile). According to the actual weight, if the body type was recognized as underweight or normal weight, it was classified as ‘no distortion of body image’.

#### 2.3.3. Weight Control

Efforts for weight control were divided into ‘no effort, weight loss, weight gain, and weight maintenance’ according to the answers to the weight control method the person tried.

#### 2.3.4. Health Behaviors

For health behaviors, ‘the number of days of moderate physical activity, vigorous physical activity, and muscle physical activity performed in the past seven days’ were collected. The level of health behaviors was classified according to the definition of the indicator in the Guidelines for the Use of Raw Data for Adolescent Health Behavior Survey [10].

Moderate physical activity was classified into ‘more than five days’, ‘less than five days’, and ‘less than five days’ of physical activity in which the heart rate increased more than usual for the past seven days or a total of 60 min or more per day. Vigorous physical activity was classified as ‘more than three days’ and ‘less than three days’ in terms of ‘more than 20 min of high-intensity physical activity such as jogging, soccer, basketball, taekwondo, mountain climbing, fast cycling, fast swimming, and carrying heavy objects in the past seven days’. Muscle physical activity was classified into ‘more than three days’ and ‘less than three days’ on the days of muscle strengthening exercises such as push-ups, sit-ups, lifting weights, dumbbells, iron bars, and parallel bars in the past seven days.

#### 2.3.5. Sedentary Behavior and Smartphone Use during Leisure Time

Leisure time sedentary behavior was collected as ‘time (min) spent sitting on average weekdays and weekends for the past seven days for activities other than learning purposes (watching TV, playing games, internet, chatting, and sitting on the go)’. The collection was divided into weekdays and weekends, and was expressed as ‘the number of minutes spent sitting outside of the average daily study purpose for seven days’, [(average time spent sitting outside of study purpose per day on weekdays × 5) + (time spent sitting on weekends other than average study purpose per day × 2)] ÷ 7, which was converted and used for the analysis. Finally, the sedentary time for non-learning activities was divided into three groups: ‘less than two hours’, ‘more than two hours–less than four hours’, and ‘more than four hours’ on average per day.

For smartphone use, ‘average time (minutes) of smartphone use per day on weekdays and weekends over the past seven days’ was collected. The collection was divided into weekdays and weekends and was expressed as ‘average smartphone use time per day (minutes) for seven days’, [(average smartphone use time per day on weekdays × 5) + (average smartphone use time per day on weekends × 2)] ÷ 7, which was used for analysis. Finally, the average smartphone usage time per day was divided into three groups: ‘less than two hours’, ‘more than two hours–less than four hours’, and ‘more than four hours’.

### 2.4. Statistical Analysis 

In the 17th KYRBWS, samples were extracted using the method of complex sampling design, so the guidelines for complex sampling analysis of the KCDC were followed. The analysis was performed using the complex samples analysis module, considering stratification variables, colony variables, and weights. For the distribution of body image distortion according to general characteristics, weight control, health behaviors, leisure time sedentary behavior, and smartphone use, the unweighted frequency and estimated ratio (weighted %) were obtained and analyzed using a Rao–Scott χ^2^ test. The factors associated with the distortion of the subject’s body image were examined by performing complex sample multiple logistic regression analysis with the statistically significant gender, grade, subjective household economic level, and subjective academic performance corrected in the Rao–Scott χ^2^ test. The program used for statistical analysis was IBM SPSS Statistics 21.0 (SPSS, Chicago, IL, USA), and statistical significance was set to a = 0.05.

## 3. Results

### 3.1. General Characteristics

A total of 41,124 adolescents were included; 19,077 (46,2%) were male, and 22,047 (53.8%) were female (Table 1). Approximately 78.1% of the study subjects found that the actual weight and the results of their subjective body image recognition were consistent, and 21.9% of the subjects analyzed their body image recognition as a distortion. There were significant differences in gender, grade, subjective academic performance, and home economy of the subjects in the body image distortion group and the no body image distortion group (Table 1, *p* < 0.05). As for the current residence type, most lived with a family (approximately 96.5%), and there were no differences in characteristics according to the type of residence (Table 1, *p* = 0.474).

### 3.2. Differences in Body Image Distortion by Weight Control

Weight control according to the subject’s body image distortion revealed significant differences in BMI and weight control efforts according to body image distortion (Table 2, *p* < 0.001). Among the adolescents with body image distortion, 24.3% had a BMI of ‘Normal’. Additionally, 40.1% of adolescents with body image distortion tried ‘Lose weight’ for weight control.

### 3.3. Differences in Body Image Distortion by Health Behaviors

An analysis of the health behaviors according to the subject’s body image distortion confirmed that there was a significant difference in moderate physical activity, vigorous physical activity, and muscle physical activity according to body image distortion (Table 3, *p* < 0.001). Among the adolescents with body image distortion, 22.8% of adolescents undertook moderate physical activity of ‘less than five days’, 23.3% of adolescents undertook ‘less than three days’ of vigorous physical activity, and 23.9% of adolescents performed muscle physical activity of ‘less than three days’.

### 3.4. Differences in Body Image Distortion According to Sedentary Behavior and Smartphone Use during Leisure Time

A significant difference in sedentary behavior and smartphone use during leisure time according to the subject’s body image distortion was found (Table 4, *p* < 0.05). Among adolescents with body image distortion, 22.0% of adolescents with sedentary behavior of ‘more than four hours’ and 22.2% of adolescents with ‘more than four hours’ using smartphones were found.

### 3.5. A Multivariate Multinomial Logistic Regression Analysis of the Factors Influencing Body Image Distortion

The factors associated with the subject’s body image distortion were identified by performing a complex samples multiple logistic regression analysis while adjusting for gender, grade, school achievement, and economic status, which were statistically significant according to the univariate analysis. The analysis confirmed that body image distortion associated with subjective body type recognition, weight control, moderate physical activity, vigorous physical activity, and sedentary behavior variables, respectively, in the state where general characteristic variables were corrected (Table 5).

The risk of recognizing subjective body type recognition as ‘little oversized’ was 8.13 times (*p* < 0.001) higher in the group with body image distortion than in the group without. For weight control, the risk of experiencing ‘no effort’, ‘weight loss’, and ‘weight gain’ was 1.04 times, 1.06 times, and 1.15 times (*p* = 0.002) higher, respectively, than those with no body image distortion. 

The risk of experiencing moderate physical activity ‘less than five days’ a week was 1.05 times (*p* = 0.022) higher in the group with body image distortion than the group without body image distortion. The risk of experiencing vigorous physical activity for ‘less than three days’ in a week was 0.93 times (*p* = 0.002) lower. 

The risk of experiencing sedentary behavior during leisure time for ‘more than two hours–less than four hours’ on average per day was 1.77 times (*p* < 0.001) higher in the group with body image distortion than the group without body image distortion, and the risk of experiencing ‘more than four hours’ on average per day was 1.58 times higher (*p* < 0.001).

## 4. Discussion

The formation of a negative body image in adolescence is a factor that affects the health of adolescents and their quality of life. Therefore, this study analyzed various factors influencing adolescents’ body image distortion. This study suggests measures to prevent body image distortion in adolescents. In this study, the BMI and subjective body image perception were compared to determine the presence or absence of body image distortion. As a result, 24.3% of adolescents recognized that they were overweight or obese despite their normal BMI (Table 2, *p* < 0.001). These results are also associated with the weight loss of adolescents. In the group without body image distortion, approximately 20.6% attempted to lose weight, whereas approximately 49.5% tried to lose weight in the group with body image distortion (Table 2, *p* < 0.001). These results in the group with body image distortion are expected to be influenced by the atmosphere of the society to which the youth belong [11,12]. Previous studies confirmed that the difference between the actual and ideal weight and body image recognition was affected by socio-cultural norms, race, age, and gender [13,14]. This is probably because the perception that a skinny body is a beautiful body shape from a young age is widespread in the youth group because of the influence of mass media and the social atmosphere that favors a skinny body [3,15]. This study confirmed that there was a difference in the presence or absence of body image distortion according to the subject’s gender, school grade, school achievement, and economic status. Among the factors influencing body image distortion, the gender difference was prominent, probably because women were more sensitive to body image distortion [16,17]. In this study, the result also confirmed that among the 41,124 adolescents included, 21.9% of the respondents who said they distorted their body image often complained of body image distortion in female students (Table 1, *p* < 0.001).

Among adolescents with body image distortion, 22.8% of adolescents undertook ‘moderate physical activity of fewer than five days’, 23.3% of adolescents performed ‘less than three days of vigorous physical exercise’, and ‘adolescents with physical muscle activity of fewer than three days’ was 23.9% (Table 3). Considering that the group with body image distortion had more experiences of trying to lose weight in this study, it can be inferred that they are choosing other methods instead of weight control through health behavior, such as physical activity. Therefore, we can consider the association between weight loss through dietary control and body image among methods for weight loss other than physical activity. According to the previous study, the probability of trying to lose weight through vomiting after eating was 1.62 times higher than regular exercise for weight loss in adolescents, confirming an inappropriate attempt to lose weight [18]. Another study found that 13.4% of adolescents fasted for weight loss, and they were performing weight loss activities, such as taking diet pills or laxatives and vomiting [19]. Excessive dietary restrictions for weight control in adolescence may cause musculoskeletal growth and sexual maturation delays or may negatively affect school life due to reduced learning ability and concentration [19]. In the results of this study, the association between inappropriate weight loss and body image distortion can be considered, so it is necessary to examine their association in depth in future studies. In addition, severe underweight was reported to increase the risk of diseases, such as decreased metabolic rate, hypotension, circulatory disorders, delayed intestinal motility or constipation, and increased mortality [20]. In addition, the possibility of body image distortion was 1.05 times (*p* = 0.022) higher in the group that performed moderate physical activity less than five days a week. Hence, inappropriate adolescent weight management attempts have a mutual effect on overall health and body image distortion. Accordingly, it is necessary to recognize the necessity of weight management through appropriate physical activities, such as the formation of consistent values for body image, balanced nutrition, and regular exercise in adolescents [21,22].

In this study, the time spent sitting and smartphone usage during the day were considered factors associated with body image distortion. As a result, there was a significant difference in sitting down and using a smartphone according to body image distortion (Table 4, *p* < 0.05). Among adolescents with body image distortion, 96.4% sat for four hours or more, and 99.1% used smartphones for four hours or more. Therefore, many adolescents spend time sitting or using a smartphone for a long time (Table 4). In particular, in the case of students sitting for more than four hours a day, the probability of body image distortion was 1.58 times higher in the analysis results, suggesting that an increase in indoor activity time was associated with the body image distortion of adolescents. According to previous studies, less physical activity is associated with more negative thoughts [23]. Body image distortion is often accompanied by cognitive distortion [24]. Therefore, cognitive distortion is expected to be associated with body image distortion because it is more likely to occur with less physical activity. Therefore, it will be necessary to provide an active environment for young people to think positively and national support for health management. On the other hand, although it was not significant, the probability of body image distortion occurring in adolescents who used smartphones for a long time was 0.81 to 0.83 times lower, which was in contrast to the expectation that smartphone use was associated with body image distortion (Table 4). This is expected to be a result reflecting the type of smartphone use in recent youth. According to a recent Korean government survey, the penetration rate of smartphones among adolescents in Korea is 95%. In other words, because most subjects use smartphones, there is a limit to claiming that the use of smartphones only affects body image distortion. Nevertheless, alternatives to prevent excessive smartphone use in adolescents are needed because the negative effects on health and mental health due to excessive use of smartphones have already been proven [25]. Physical activity is recommended to prevent overdependence on smartphones among adolescents. A previous study confirmed the association between physical activity and smartphone dependence among adolescents and that physical activity effectively reduced smartphone usage time, especially in team activities [26]. This would be a necessary countermeasure even when considering that the possibility of body image distortion was high in the group that spent considerable time indoors in this study. Therefore, it will be necessary to actively introduce a program that includes team activity-based physical activity to form no distortion of adolescents’ body image.

This study is meaningful as a source of primary data for the importance of consistent body shape recognition in adolescence by analyzing factors affecting body image distortion, which is a concern in adolescents, and for preparing alternatives for this. On the other hand, this study was a one-off survey using the results of an online youth survey, and there was a limitation in inferring a causal association between each variable. Therefore, a well-organized further study should be conducted based on the results found in this study to overcome these limitations. A systematic intervention program for no distortion of body image formation in adolescence should be developed in the future.

## 5. Conclusions

Adolescents’ weight control, physical activity, and sedentary behavior were found to be associated with the distortion of adolescents’ body image. Therefore, it is thought that institutional improvement and the development of a systematic intervention program are necessary to form an undistorted body image among adolescents.

## Figures and Tables

**Table 1:** Differences in body image distortion by general characteristics (*n* = 41,124).

Variables	Categories	Total	Body Image Distortion		Rao–Scott χ^2^	*p*
		*n* (%)	No	Yes		
			*n* (%)	*n* (%)		
Gender	Boys	19,077 (46.2)	16,189 (84.9)	2888 (15.1)	988.65	<0.001
	Girls	22,047 (53.8)	15,888 (72.2)	6159 (27.8)		
Grade	Middle school	22,639 (51.4)	17,835 (78.7)	4804 (21.3)	10.51	0.024
	High school	18,485 (48.6)	14,242 (77.4)	4243 (22.6)		
School achievement	High	5447 (13.1)	4427 (81.7)	1020 (18.3)	127.66	<0.001
	Middle high	10,426 (25.3)	8281 (79.4)	2145 (20.6)		
	Middle	12,771 (31.2)	10,020 (78.5)	2751 21.5)		
	Middle low	8681 (21.2)	6526 (75.2)	2155 (24.8)		
	Low	3799 (9.2)	2823 (74.3)	976 (25.7)		
Economic status	High	4397 (10.7)	3575 (81.0)	822 19.0)	79.74	<0.001
	Middle high	11,908 (29.8)	9411 (78.9)	2497 (21.1)		
	Middle	20,446 (49.3)	15,897 (77.8)	4549 (22.2)		
	Middle low	3648 (8.6)	2665 (73.3)	983 (26.7)		
	Low	725 (1.6)	529 (74.3	196 (25.7		
Residential type	Family	39,395 (96.5)	30,703 (78.0)	8692 (22.0)	12.97	0.474
	Relatives	186 (0.4)	146 (77.3)	40 (22.7)		
	Lodging	182 (0.4)	140 (76.6)	42 (23.4)		
	Dormitory	1235 (2.4)	984 (79.7)	251 (20.3)		
	Childcare facilities	126 (0.2)	104 (82.6)	22 (17.4)		

All values are presented as unweighted numbers (weighted %). *p*-values were obtained using a Rao–Scott χ^2^ test.

**Table 2:** Differences in body image distortion by weight control (*n* = 41,124).

Variables	Categories	Total	Body Image Distortion		Rao–Scott χ^2^	*p*
		*n* (%)	No	Yes		
			*n* (%)	*n* (%)		
BMI	Low weight	3847 (9.7)	3847 (100)	0 (0)	2100.60	<0.001
	Normal	37,277 (90.3)	28,230 (75.7)	9047 (24.3)		
Weight control	No effort	21,061 (51.6)	17,745 (84.2)	3316 (15.8)	3629.08	<0.001
	Lose weight	11,068 (26.8)	6594 (59.9)	4474 (40.1)		
	Gain weight	3785 (9.1)	3708 (97.8)	77 (2.2)		
	Maintain weight	5210 (12.5)	4030 (77.2)	1180 (22.8)		

All values are presented as unweighted numbers (weighted %). *p*-values were obtained using a Rao–Scott χ^2^ test.

**Table 3:** Differences in body image distortion by health behaviors (*n* = 41,124).

Variables	Categories	Total	Body Image Distortion		Rao–Scott χ^2^	*p*
		*n* (%)	No	Yes		
			*n* (%)	*n* (%)		
Moderate physical activity	<5 days/wk	35,020 (85.8)	26,972 (77.2)	8048 (22.8)	122.31	<0.001
	≥5 days/wk	6104 (14.2)	5105 (83.5)	999 (16.5)		
Vigorous physical activity	<3 days/wk	28,795 (71.2)	(76.7)	6755 (23.3)	112.62	<0.001
	≥3 days/wk	12,329 (28.8)	10,037 (81.4)	2292 (18.6)		
Muscle physical activity	<3 days/wk	31,711 (77.6)	24,075 (76.1)	7636 (23.9)	344.79	<0.001
	≥3 days/wk	9413 (22.4)	8002 (84.9)	1411 (15.1)		

All values are presented as unweighted numbers (weighted %). *p*-values were obtained using a Rao–Scott χ^2^ test.

**Table 4:** Differences in body image distortion according to the time of sedentary behavior and smartphone use during leisure time. The values are presented as the mean± standard error or unweighted number (weighted %).

Variables	Categories	Total	Body Image Distortion		Rao–Scott χ^2^	*p*
		n (%)	No	Yes		
			n (%)	n (%)		
Time of sedentary behavior	<2 h/day	955 (2.4)	766 (80.9)	189 (19.1)	6.31	0.043
	2–4 h/day	889 (2.2)	702 (79.8)	187 (20.2)		
	≥4 h/day	38,370 (95.5)	29,930 (78.0)	8440 (22.0)		
Time of using smartphones	<2 h/day	98 (0.2)	80 (85.2)	18 (14.8)	6.65	0.027
	2–4 h/day	375 (0.9)	312 (81.8)	63 (18.2)		
	≥4 h/day	39,107 (98.9)	30,429 (77.8)	8678 (22.2)		

All values are presented as unweighted numbers (weighted %). The total number of some variables is different due to missing values. *p*-values were obtained using a Rao–Scott χ^2^ test.

**Table 5:** Multivariate multinomial logistic regression analysis of the factors influencing body image distortion.

Variables	Categories	Body Image Distortion		
		OR	95% CI	*p*
Subjective body type recognition	Very oversized	2.95	2.49–3.49	<0.001
	Little oversized	8.13	7.37–8.97	
	Normal	0.20	0.18–0.21	
	Little undersized	0.29	0.26–0.31	
	Very undersized	1		
Weight control	No effort	1.04	0.99–1.08	0.001
	Lose weight	1.06	1.00–1.11	
	Gain weight	1.15	1.07–1.24	
	Maintain weight	1		
Moderate physical activity	<5 days/wk	1.05	1.01–1.10	0.022
	≥5 days/wk	1		
Vigorous physical activity	<3 days/wk	0.93	0.89–0.97	<0.001
	≥3 days/wk	1		
Muscle physical activity	<3 days/wk	1.04	1.00–1.08	0.078
	≥3 days/wk	1		
Time of sedentary behavior	≥4 h/day	1.58	1.42–1.77	<0.001
	2–4 h/day	1.77	1.54–2.03	
	<2 h/day	1		
Time of using smartphones	≥4 h/day	0.81	0.64–1.01	0.179
	2–4 h/day	0.83	0.64–1.07	
	<2 h/day	1		

OR, odds ratio; CI, confidence interval. OR was obtained by a multiple logistic regression analysis adjusted for gender, grade, school achievement, economic status, and BMI.

## Data Availability

Not applicable.
